# Model fidelity and team members’ experiences of assertive community treatment in Norway: a sequential mixed-methods study

**DOI:** 10.1186/s13033-019-0321-8

**Published:** 2019-10-16

**Authors:** Sigrun Odden, Anne Landheim, Hanne Clausen, Hanne Kilen Stuen, Kristin Sverdvik Heiervang, Torleif Ruud

**Affiliations:** 1 Norwegian National Advisory Unit On Concurrent Substance Abuse and Mental Health Disorders, Innlandet Hospital Trust, Brumunddal, Norway; 2 Department of Public Health, Inland Norway University of Applied Sciences, Elverum, Norway; 3 Dept. of Research & Development, Mental Health Services, Akershus University Hospital, Lørenskog, Norway; 4 Mental Health Services, Akershus University Hospital, Lørenskog, Norway; 5 Institute of Clinical Medicine, University of Oslo, Oslo, Norway

**Keywords:** Assertive community treatment, Model fidelity, Team members, Tool for Measurement of Assertive Community Treatment (TMACT), Implementation

## Abstract

**Background:**

Assertive community treatment (ACT) is an evidence-based treatment for people with severe mental illness, and this model is used widely throughout the world. Given the various adaptations in different contexts, we were interested in studying the implementation and adaptation of the ACT model in Norway. The first 12 Norwegian ACT teams were established between 2009 and 2011, and this study investigated the teams’ model fidelity and the team members’ experiences of working with ACT.

**Methods:**

To investigate implementation of the ACT model, fidelity assessments were performed 12 and 30 months after the teams started their work using the Tool for Measurement of Assertive Community Treatment (TMACT). Means and standard deviations were used to describe the ACT teams’ fidelity scores. Cohen’s effect size d was used to assess the changes in TMACT scores from the first to second assessment. Qualitative focus group interviews were conducted in the 12 teams after 30 months to investigate the team members’ experiences of working with the ACT model.

**Results:**

The fidelity assessments of the Norwegian teams showed high implementation of the structural and organizational parts of the ACT model. The newer parts of the model, the recovery and evidence-based practices, were less implemented. Four of the six subscales in TMACT improved from the first to the second assessment. The team members experienced the ACT model to be a good service model for the target population: people with severe mental illness, significant functional impairment, and continuous high service needs. Team members perceived some parts of the model difficult to implement and that it was challenging to find effective ways to collaborate with existing health and social services.

**Conclusion:**

The first 12 Norwegian ACT teams implemented the ACT model to a moderate degree. The ACT model could be implemented in Norway without extensive adaptations. Although the team members were satisfied with the ACT model, especially the results for their service users, inclusion of the ACT team to the existing service system was perceived as challenging.

## Introduction

Assertive community treatment (ACT) was developed in the 1970s as a “hospital without walls” to achieve better functioning in the community for people with severe mental illness [1]. ACT is a model for comprehensive, integrated, community-based services based on a multidisciplinary team that provides psychiatric treatment, social services and rehabilitation. This model is used widely throughout the world and has been well documented by research [2–5]. It has been acknowledged as evidence-based treatment [6], and the last Cochrane review found that intensive case management, which includes ACT, is more effective in ameliorating many outcomes relevant to people with severe mental illness than non-intensive case management and standard care [7]. This review found that the more intensive case management is adherent to the ACT model, the better it is at decreasing time in hospital [7].

Positive results from randomized trials of ACT in the USA and Australia were not replicated in the United Kingdom and the Netherlands, especially regarding reduction in hospitalization [8–11]. The inconsistent results of studies in US and UK have been explained by differences in the contexts in which the trials were conducted [12, 13]. The reduction in hospitalization is less successful in contexts where hospital use is already low [12]. In UK there was reason to doubt the value of investing in high model fidelity when ordinary services like community mental health teams share most of the organizational aspects of ACT and deliver equal outcomes [13].

Because ACT outcomes vary between different health care systems, it is of great research interest to study ACT adaptation in different countries with different cultural contexts, with different amounts of services in both primary and secondary health care and different population density with different travel distances. Innovation studies have shown that political, organizational, economic and structural conditions, as well as characteristics of the users and providers, can influence the implementation of new models [14, 15]. New service models often needed to be tailored to local conditions and resources [16, 17]. ACT is a complex intervention involving several activities and many interpersonal interactions. The UK Medical Research Council has shown that complex interventions work best when tailored to local circumstances rather than being standardized completely [17].

The implementation of the ACT model in different contexts has produced different variants of the ACT model. The model has been extended and adapted to different groups and contexts. The flexible ACT (FACT) model [18, 19], is a Dutch adaptation of ACT that combines ACT with less intensive individualized case management during more stable periods. Most rural ACT programmes have made significant modifications to the ACT model. Examples include specialized ACT teams for consumers involved with the criminal justice system [20, 21] and for consumers with substance use disorders in the Netherlands [22]. ACT has been modified to suit people undergoing first-episode psychosis [23, 24] and homeless people [25, 26]. The flexibility of ACT has permitted many adaptations and remains relevant in different service systems [27].

Fidelity scales define the critical ingredients of ACT and measure to what extent the elements are implemented. In addition to ensuring that services are provided in accordance with the model, fidelity measurement provides guidelines for replication as well as potential for defining model adaptations [28]. The Dartmouth Assertive Community Treatment Scale (DACTS) [29] is used widely to measure fidelity to the ACT model. A new standard for ACT teams in the USA includes evidence-based practice and recovery orientation [30]. To meet the development in the mental health services, especially in evidence-based practices and recovery orientation, a new fidelity measurement based on the DACTS was developed—the Tool for Measurement of Assertive Community Treatment (TMACT) [31].

The Norwegian Directorate of Health has supported the implementation and evaluation of the ACT model since 2009 [32]. The directorate provides financial incentives, a Norwegian handbook on the principles of ACT, a training programme with seminars for team members and a research-based evaluation of the project [33]. Twelve ACT teams were established between 2009 and 2011 to test the ACT model in different local contexts. At the time of submission of this article four of the 12 teams are still ACT teams, five teams have changed from ACT team to FACT team and three teams have closed down.

Norway differs in many ways from the USA and the UK, where most studies of ACT have been conducted. Norway has a population of five million who live in about 400 municipalities, half of them with less than 5000 inhabitants [34]. The public social policy has a large scope and is aimed to meet the basic needs of all citizens regardless of their economic position [35]. Norway has well-developed health and social services within the framework of the Nordic welfare state [36]. Unlike the UK, it does not have community mental health teams. Mental health services are divided into two organizational levels: primary health services, for which the municipality is the responsible unit, and specialized health services run by health trusts owned by the government through regional health authorities [35].

The large scope of services makes both horizontal and vertical co-ordination challenging and has resulted in fragmented services and a lack of continuity for service users [37]. The division between the services and the fragmentation of the services were important reasons for implementing the ACT model in Norway. The essential goals were to improve collaboration between services and create better, more comprehensive and integrated services for people with substantial and complex problems, including substance use disorders and mental illness, who need help from a variety of services [38]. The Norwegian ACT teams were established as collaborations between the municipalities and local community mental health centres in the specialist health services.

Since the ACT model has different adaptations and varying outcomes, it is of great interest to determine whether the ACT model is relevant in Norway and how it can be adapted into a country with well-developed health and social services, with multiple and fragmented services and in regions with low population density.

The aim of this paper was to investigate the implementation and adaptation of the ACT model in Norway. The following two research questions were addressed.

1. What was the model fidelity for the Norwegian ACT teams?
2. What were the team members’ experiences of working with the ACT model?

To answer the first research question, we focused on model fidelity to the different parts of the ACT model, variations between teams and changes in fidelity scores from 12 to 30 months after the teams began. To answer the second research question, we focused on the challenges and advantages experienced by team members working with the ACT model and their experience of the collaboration with other services.

## Methods

### Design

This study was part of a research-based evaluation of the first 12 ACT teams in Norway [39]. To gain a broader understanding of the ACT implementation and adaptation, a sequential mixed-methods design was used. This included quantitative fidelity assessments of 12 teams at 12 months and 30 months after the teams started. A cross-sectional qualitative study was conducted to investigate the team members’ experiences of working with the ACT model.

### Context

The 12 first ACT teams in Norway were established between December 2009 and February 2011. They were located in different parts of Norway, in both urban and rural areas; some operated in the largest cities, but most teams were in smaller towns. The largest catchment area had more than 100 000 inhabitants, and the smallest about 40 000 inhabitants. The teams differed in the number of service users, number of service providers, range of catchment area and local organization. The number of team members ranged between 4.8 and 11.9 full-time equivalents, and the ratio of consumers to staff ranged between 3 and 11. Half of the teams contained two or more municipalities in their catchment area. All teams were established as collaborations between the municipalities and the local community mental health centre (CMHC) in the specialist health services. Most teams were organizationally anchored in the CMHC and all team members were employed by the CMHC. Some teams had a mix of staff employed in the municipality and staff employed in the CMHC.

### Sample

The sample for the fidelity measurement comprised the first 12 ACT teams in Norway, which represented the entire population of ACT teams in Norway. They were all newly established teams. The sample for the focus group interviews were all team members on duty in the 12 teams. 72 team members from the 12 ACT teams participated in focus group interviews; four team members from the smallest team and 11 from the largest team. These participants represented the different occupational groups in the teams: nurses, social workers, social educators, psychologists and psychiatrists. The vast majority of these participants had considerable professional experience working with patients with severe mental illness.

### Data collection and measurement

#### ACT fidelity

TMACT version 1 was used to assess the model fidelity for the 12 ACT teams. TMACT is a fidelity scale with 47 items divided into six subscales: (1) Operation and Structure, (2) Core Team, (3) Specialist Team, (4) Core Practices, (5) Evidence-Based Practices and (6) Person-Centered Planning and Practices [26]. The Operation and Structure subscale contains 12 items assessing the team processes and team organization, such as who and how many individuals the team is to serve and the daily team meeting (attendance, frequency and quality). The core team comprises of team leader, nursing staff and psychiatric care provider. Seven items in the Core Team subscale assess their positions, their role within the team and the services they provide to consumers. The specialist team comprises of the substance use specialist, vocational specialist and peer specialist. Eight items in the Specialist Team subscale assess their positions, their role within the team and the services they provide to consumers. The Core Practice subscale includes eight elements that assess core ACT services such as working assertively outreach with intensive and frequent contacts with consumers and the team’s full responsibility for psychiatric services and rehabilitation services. Evidence-based practice including integrated dual disorder treatment, supported employment, wellness management and recovery, supportive housing and family psycho-education are assessed across eight items. The Person-Centered Planning and Practice subscale include four items that facilitate recovery by enhancing consumer self-determination (Table 1 shows the items in the TMACT scale).

**Table 1:** TMACT subscales and items at 12 months and 30 months (12 teams)

	T1 (12 months)		T2 (30 months)		Cohen’s d^a^
	Mean	SD	Mean	SD	
TMACT total score	3.3	0.32	3.6	0.28	0.83
Operations and Structure (OS) Subscale	3.9	0.41	4.1	0.36	1.59
OS1 Low Ratio of Consumers to Staff	5.0	0.00	4.9	0.29	− 0.29
OS2 Team Approach	3.3	1.29	3.2	1.47	− 0.16
OS3 Daily Team Meeting (Frequency and Attendance)	3.8	1.59	4.2	1.34	0.19
OS4 Daily Team Meeting (Quality)	4.1	1.00	4.7	0.65	0.59
OS5 Program Size	2.7	1.83	3.3	1.82	0.47
OS6 Priority Service Population	4.3	1.23	4.7	1.16	0.51
OS7 Active Recruitment	2.7	0.49	2.8	1.03	0.15
OS8 Gradual Admission Rate	4.7	0.49	4.8	0.39	0.29
OS9 Transition to Less Intensive Services	3.1	0.67	4.1	0.90	1.17
OS10 Retention Rate	4.6	0.67	4.8	0.62	0.29
OS11 Involvement in Psychiatric Hospitalization Decisions	4.7	0.65	4.8	0.45	0.12
OS12 Dedicated Office-Based Program Assistance	3.4	1.78	3.4	1.62	0.00
Core Team (CT) Subscale	3.6	0.55	3.9	0.46	0.68
CT1 Team Leader on Team	2.5	1.24	3.0	1.21	0.40
CT2 Team Leader is Practicing Clinician	3.3	0.78	3.3	0.99	0.00
CT3 Psychiatric Care Provider on Team	4.3	1.23	4.5	0.91	0.29
CT4 Role of Psychiatric Care Provider in Treatment	2.6	1.38	3.3	1.06	0.68
CT5 Role of Psychiatric Care Provider within Team	3.3	0.99	3.7	0.65	0.68
CT6 Nurses on Team	5.0	0.00	5.0	0.00	0.00
CT7 Role of Nurses	4.3	0.62	4.5	0.52	0.55
Specialist Team (ST) Subscale	2.5	0.62	2.5	0.70	0.12
ST1 Substance Abuse Specialist on Team	3.5	1.45	3.7	1.23	0.10
ST2 Role of Substance Abuse Specialist in Treatment	3.2	1.59	3.7	1.07	0.43
ST3 Role of Substance Abuse Specialist within Team	3.3	1.37	3.8	1.22	0.34
ST4 Vocational Specialist on Team	2.4	1.31	1.9	0.9	− 0.36
ST5 Role of Vocational Specialist in Employment Services	2.3	1.16	2.3	1.14	− 0.11
ST6 Role of Vocational Specialist within Team	2.9	1.09	2.6	1.24	− 0.29
ST7 Peer Specialist on Team	1.2	0.39	1.2	0.58	0.00
ST8 Role of Peer Specialist	1.3	0.45	1.2	0.58	− 0.16
Core Practices (CP) Subscale	3.6	0.40	3.7	0.37	0.25
CP1 Community-Based Services	5.0	0.00	5.0	0.00	0.00
CP2 Assertive Engagement	4.6	0.52	4.9	0.29	0.68
CP3 Intensity of Service	3.8	0.75	3.3	0.89	− 0.62
CP4 Frequency of Contact	2.9	0.79	2.8	0.62	− 0.29
CP5 Frequency of Contact with Natural Supports	2.8	0.75	2.3	0.78	− 0.42
CP6 Responsibility for Crisis Services	1.4	0.52	1.7	0.49	0.55
CP7 Full Responsibility for Psychiatric Services	3.8	1.34	4.7	0.65	0.75
CP8 Full Responsibility for Psych. Rehabilitation Services	4.2	1.03	4.5	0.67	0.34
Evidence-Based Practices (EP) Subscale	2.9	0.69	3.4	0.38	0.90
EP1 Full Responsibility for Dual Disorders Treatment	3.0	1.28	4.0	1.04	0.89
EP2 Full Responsibility for Vocational Services	3.3	1.56	3.6	1.56	0.11
EP3 Full Responsibility for Wellness Man. and Recovery	1.0	0.00	1.1	0.29	0.29
EP4 Integrated Dual Disorders Treatment Model	3.0	0.95	4.0	0.43	1.05
EP5 Supported Employment Model	2.3	0.65	2.5	0.91	0.23
EP6 Engagement & Psychoeducation with Natural Supports	3.8	1.12	4.5	0.80	0.68
EP7 Empirically Supported Psychotherapy	2.3	1.49	3.2	1.19	0.92
EP8 Supportive Housing Model	4.3	1.06	4.3	1.06	0.00
Person-Centered Planning and Practices (PP) Subscale	2.9	0.52	3.6	0.59	0.75
PP1 Strengths Inform Treatment Plan	2.6	0.90	3.3	0.99	0.78
PP2 Person-Centered Planning	1.3	0.49	1.8	0.97	0.53
PP3 Interventions Target Broad Range of Life Domains	3.6	1.00	4.3	1.14	0.36
PP4 Consumer Self-Determination and Independence	4.1	0.67	4.8	0.45	0.86

^a^Interpretation of Cohen’s d: 0.2 indicates a small effect, 0.5 a moderate effect and 0.8 a large effect [41]

The first fidelity assessment was done 12 months after a team started, in December 2010 for the first team and in February 2012 for the last team. The second fidelity assessment was done 30 months after a team started, in June 2012 for the first team and in August 2013 for the last team. The fidelity assessments were conducted by a six-member research group (the authors of this article), which comprised three groups of two people who had responsibility for the fidelity assessments for four ACT teams each. The fidelity assessments were performed according to the TMACT manual [40]. The six-member research group was trained by the American developers of the TMACT (Monroe-De Vita and Teague). The manual contains detailed guidelines and rules on data collection and rating.

At each site, two fidelity assessors had a 2-day onsite visit, during which they interviewed team members and observed team processes. Before the team visit, the team completed questionnaires to provide information about the team and services. The fidelity assessments were based on the following data sources obtained from each team: semi-structured interviews with all team members (seven interviews, one with each group of team members who shared a specific role); interviews with service users; survey about the team and team members; spread sheet with data about the service users and the services they received from the team; observation of a daily team meeting and a treatment planning meeting; observation of community/home visits with one to two team members as they worked with service users; and chart reviews (random selection of 10 service users).

#### Focus group interviews

To investigate the team members’ experiences with the ACT model, qualitative focus group interviews were conducted for all 12 ACT teams. Interviews were conducted from March to November 2013, which was about 30 months after the teams had started and after the last fidelity assessment. The focus groups were moderated by the same research group members who conducted the fidelity assessments. The first author participated in all 12 interviews. Each interview occurred on the same day that the research group gave feedback to the team from the last fidelity assessment. The focus group interviews followed an interview guide that focused on the team members’ views of the ACT team’s fidelity scores and their experiences with the ACT model.

The moderator ensured that all main issues were covered but had no strict management of the interviews. The interview form was semi-structured, but the ACT team members were encouraged to express their views and discuss their experiences freely. The interviews took the form of a conversation between the staff based on some general questions asked by the moderator. The focus group interviews lasted from 1.5 to 2 h for each team.

### Data analysis

#### ACT fidelity

Each fidelity assessor rated the fidelity scale independently before comparing and discussing these ratings with his/her partner to reach a consensus rating. The preliminary assessments were presented to the team, the ratings were discussed and a final report was then completed. The whole research group also reviewed and discussed the scores for all 12 teams to ensure that the fidelity assessments were performed in the same way for all teams.

The 47 items in the TMACT scale were rated on a 5-point scale from 1 (not implemented) to 5 (fully implemented). The total mean TMACT scores were interpreted according to the TMACT manual as follows: not implemented (1.0–2.4), low fidelity (2.5–3.1), moderate fidelity (3.2–3.7), high fidelity (3.8–4.3) and exemplary fidelity (4.4–5.0).

Statistical analyses were conducted using IBM SPSS Statistics 23. Means and standard deviations (SDs) were used to describe the ACT teams’ fidelity scores. Cohen’s effect size d was calculated to assess the changes in fidelity scores from 12 to 30 months; however, we did not test for significant differences because this was a population study. A rule of thumb for interpreting Cohen’s d results is that 0.2 indicates a small effect, 0.5 a moderate effect and 0.8 a large effect [41].

#### Focus group interviews

The focus group interviews were audio recorded and transcribed. The data were processed and analysed according to the principles for qualitative content analysis and systematic text condensation [42]. The transcript was thematically systematized and coded; most of the codes were from the interview guide, and some came from the data. The interviews resulted in extensive data material, and a small part of the synthesized text is presented after cross-case comparison. The most relevant data that answered the research questions were selected.

## Results

### ACT fidelity

The Norwegian teams’ fidelity according to the TMACT is presented in Table 1. The TMACT scores revealed moderate implementation of the ACT model. The mean total score was 3.3 at the 12-month assessment and 3.6 at the 30-month assessment. Four of six subscales had improved from the first to the second assessment. The subscales *Evidence*-*Based Practices* and *Person*-*Centered Planning and Practices* were the parts of the model with the greatest changes, and these improved from low to moderate fidelity. The Subscales of *Operation and Structure* and *Core Team* improved from moderate to high fidelity from 12 to 30 months. Cohen’s effect sizes for the changes from 12 to 30 months were large for the TMACT total score and for the subscales of *Operation and Structure, Evidence*-*Based Practices* and *Person*-*Centered Planning and Practices*. The effect sizes of the changes from 12 months to 30 months were small for the *Core Practice* and the *Specialist Team* subscales.

After 30 months, the subscales of *Operation and Structure* and the *Core Team* had high implementation in the Norwegian ACT teams. The newer parts of the model were implemented to a lesser degree. The subscales *for Evidence*-*Based Practices* and *Person*-*Centered Planning and Practice* had moderate fidelity scores and the *Specialist Team* subscale had a low fidelity score.

For the subscale of *Operation and Structure* we found that the Norwegian teams recruited a priority service population in accordance with the model. This meant that they included service users who had a severe mental illness (schizophrenia, schizoaffective disorder, other psychotic disorder, or bipolar disorder), an impaired level of everyday functioning in addition to a need of long-term and comprehensive follow-up by mental health and social welfare services. The teams had low caseload, included service users gradually and retained a high percentage of their service users. The teams were also involved in the service users’ admissions and discharges. For the *Core Team* subscale, the teams included sufficient numbers of nurses, psychiatrists and team leaders, and the nurses performed their role in accordance with the model. For the *Specialist Team* subscale, the teams had not included peer specialists or vocational specialists in the teams. The item for the substance abuse specialist had moderate fidelity. For the *Core Practices* subscale, the teams worked almost exclusively in the community and used several techniques to engage their service users. They assumed responsibility for providing psychiatric services and psychiatric rehabilitation services, but they did not assume responsibility for crisis services expected in the model. The *Frequency of Contact with Natural Supports* subscale also had low fidelity. For the *Evidence*-*Based Practice* subscale, the teams had high fidelity on engagement with service users’ family and natural network, supportive housing and dual disorders treatment. However, they did not meet the requirements for wellness management. For the *Person*-*centred Planning and Practices* subscale, the teams promoted the service users’ independence and self-determination, and they specified interventions that targeted a range of life domains. However, they had not implemented the person-centred planning in the treatment planning process according to the model.

**Fig. 1:**
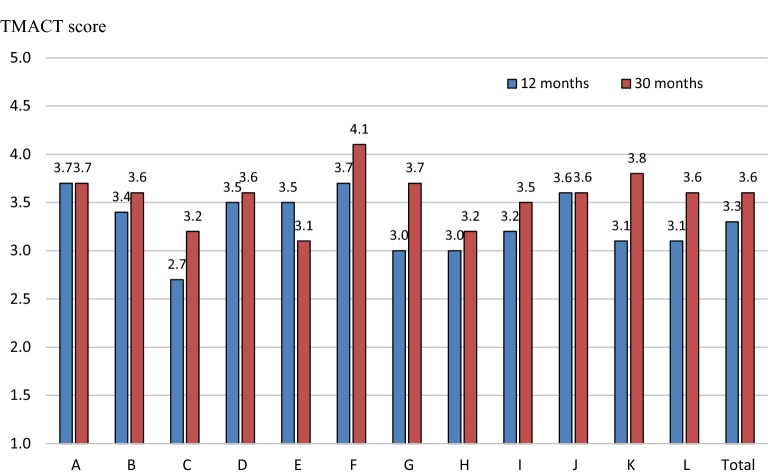
TMACT total mean scores for the 12 Norwegian ACT teams at 12 months and 30 months

Comparison of the 12 Norwegian teams showed that the mean of the TMACT total score ranged from 3.0 to 3.7 at the 12-month assessment (see Fig. 1). Six teams had low fidelity and six teams had moderate fidelity. At the 30-month assessment, the TMACT total scores ranged from 3.1 to 4.1. At the second assessment, two teams had high fidelity, one had low fidelity and nine teams had moderate total fidelity.

### Experiences with the ACT model

#### Advantages of working with the ACT model

Overall, the team members were satisfied with the ACT model. They perceived that various parts of the model are important for achieving good results for the service users. Outreach services were highlighted because they provided opportunities to focus on the service users’ resources and help them to cope with everyday life. The high intensity and frequency of contact between team members and service users was perceived as important because it inspired the service users’ confidence in the staff and provided predictability and structure in the daily life of the service users. The team members also highlighted that the frequency of contact also meant that service users and team members had more time to see each other in different ways than in traditional, office-based services.

The team members emphasized the importance of continuity and long-term processes, and service users’ involvement. The team members stated that attending to the service users’ needs provided an important type of support. Interdisciplinary teams were regarded positively because they provided different approaches to problems. The skills and professional perspectives of the different team members complemented each other and allowed the teams to produce creative and practical solutions that benefitted the service users.

Building good relations with the service users was the first thing mentioned by the team members when they were asked about their experiences of working with the ACT model. They thought they had succeeded in building good, trusting relationships and that these ensured better opportunities for them to provide treatment. Team members said that they had managed to establish and maintain contact with service users who had withdrawn from services. The good relationships were thought to be the result of the teams’ flexibility and their broad range of services, through which they were able to offer, in addition to treatment, practical help and support with everyday activities, housing, money management and access to activities. The following is a quote from a team member.

*We’ve had both the time and resources necessary to come in and build a relationship, and that relationship we may now use constructively (…) so now we have some options if we are to save his life.*

Despite differences between teams, the following common positive experiences were highlighted by the team members: home-based services, intensity and frequency of contacts, service user involvement, transdisciplinary team approach, flexibility and a wide range of services.

#### Challenges from working with the ACT model

Many staff members indicated that it was unclear to them how the specialist functions (vocational specialist, substance abuse specialist and peer specialist) should be performed, and they felt a need for more training in these functions. Most specialists worked as generalists. Supported employment was the model requirement that most team members found impossible to fulfil. This is linked to the Norwegian Labour and Welfare Administration, which is responsible for the service users’ social benefits and pensions. The ACT team members did not perceive this government department as being supportive to find competitive work for the service users. They noted that a common attitude linked to the rules and regulations was, *“If you do ordinary work, you will lose your pension!”* The team members mentioned supported employment as an item they want to improve. However, they often ended up providing specialized work and meaningful activities for their service users, and not competitive work as required in the ACT model.

There were no ambitions among the Norwegian teams to fulfil the ACT requirement of 24-h coverage. Most team members did not find it necessary, and they regarded alternative solutions as sufficient. These solutions involved collaboration with other services, such as crisis resolution teams to provide support to the service users at night and on weekends. Intensity of services was regarded as an important part of the model by the team members, but most of them claimed that the model has unrealistic requirements. Three face-to-face-contacts per week per service user were considered to be unrealistic given the time required for travelling in addition to contact with users. Some team members emphasized that one contact per week was sufficient for certain individual service users depending on the needs. The team approach was considered to be important, although some teams limit the number of team members providing face-to-face contacts with particular service users.

#### Collaboration with other services

The ACT staff often highlighted that it was important that the specialist services and the primary services co-operated in the ACT team.

*What’s unique is that we come from both levels*—*from the hospital and the municipality. We have both*—*that’s what makes ACT successful!*

There was consensus among the team members that ACT teams must co-operate with other parts of the service system. The ACT team was often said to have a co-ordinating function between different services; this was referred to by team members as being a “bridge builder” or “translator”. The teams’ experience of their co-operation with other services differed. Some teams experienced good co-operation with specialist health services and challenges with municipal services, whereas it was the opposite for other teams. Some teams noted that it was challenging that their collaborator (also their co-owner) wanted them to provide services to a larger target group than the target group described in the ACT model.

For some services, team members considered that collaboration with other parts of the service system was necessary because of legal regulations, whereas for other services, collaboration was considered to benefit the service users. The teams had to co-operate with the municipality regarding housing for their service users. For services such as home help and home nursing help with medication, team members had different experiences and views about the division of roles and functions between the services. Most team members found it reasonable that the primary services were involved in some services for ACT service users. One team member said, “*We can clean now and then, but we’re not a regular home help service”.* ACT teams were considered to be part of a service system and not an isolated service. Another team member said, “*It is best for the users to be connected to some extent to other service providers*”. They emphasized that the ACT team gained an overall view and assumed responsibility for their service users, even if they did not provide all services. Reflecting on their collaboration with external partners, team members believed it important to develop a shared understanding of the ACT model and of who should provide which services.

## Discussion

### ACT fidelity

We focused on the implementation of the ACT model and found that the Norwegian ACT teams had moderate implementation of the model. The new parts of the model, recovery orientation and evidence-based practices, were less implemented than the parts usually captured in the earlier fidelity scales such as the DACTS. The Norwegian teams were newly developed teams that were implementing a new practice. It was therefore expected that the more traditional and typical parts would have been implemented before the new and more special and demanding parts of the model, which also were less emphasized in the training of the teams. Essential elements of the model showed high fidelity, such as the structural and organizational components, which have been found to be important in reducing the hospitalization of ACT clients [12].

The fidelity assessments of the Norwegian teams showed higher fidelity at the second than at the first assessment. Improvement in fidelity over time has been shown in several other studies [40, 43, 44]. The low implementation of specialist staff in the ACT model had some contextual explanations. For example, there are no specialized educational programmes for vocational specialists, and peer support was not an integrated part of the mental health services in Norway when the evaluation was conducted. The team leaders were usually nurses who had completed continuing education but not a master’s degree, which is a model requirement. Psychologists often provided important resources in the Norwegian teams, but their specialist role is not described in the ACT model.

Our study is one of the first to use the fidelity instrument TMACT. Because this is a relatively new instrument, there are few other studies for comparison. In pilot testing of the TMACT in the United States, the fidelity scores for the 10 teams in Washington State were higher than those for the 12 Norwegian teams. The mean score for TMACT total was 4.2 after 18 months in the Washington teams [31] and 3.6 after 30 months in the Norwegian teams. The 10 ACT teams in Washington State had the same pattern of fidelity scores as the Norwegian teams. TMACT ratings were higher for core ACT practices than for recovery practices and evidence-based practices [31]. Even though some findings in the Norwegian study can be explained by national factors as education of health professionals and organisation of mental health care in Norway, the pattern for the Washington State teams and the Norwegian teams suggest additional cross-cultural explanations. Fidelity assessments in other countries, like Canada, also showed lower fidelity ratings in the areas of recovery, specifically for employment and substance abuse, and staff working in these areas than for core ACT practices [45].

### Experiences with the ACT model

The Norwegian team members were satisfied with the ACT model despite the challenges of fulfilling some parts of the model such as supported employment, crisis services and frequency of contacts. They thought that the ACT is a good model for the target population. They were primarily satisfied with the results for their service users, and they perceived that the model worked and that service users were doing better in many ways. A Canadian study reported that ACT staff were largely positive about their involvement with the services and especially valued the opportunity to develop close, trusting relationships with service users; however, some ACT standards were found to be ambiguous and subsequently difficult to implement [46].

The positive experiences were supported by results from the research-based evaluation of the 12 teams, which found improvements in several areas for the patients in a 2-year follow-up [39]. A study of the service users revealed a high level of satisfaction with the service [47]. A study of inpatient service use within the service showed that participants spent significantly fewer days in hospital in the 2 years during ACT compared with the 2 years before enrolment in ACT [48].

The results from both the focus group interviews and fidelity assessments showed that the ACT model was implemented in Norway without the need for extensive adaptations of the

ACT model. This suggests that the comprehensive approach taken by ACT teams responded to a need in the Norwegian service system for more intensive and comprehensive services.

#### Collaboration with other services

A challenge when adapting the ACT model in Norway relates to the organizational context of several different services in the health and welfare system, which is organized at two levels and has different legislation and financing. The purpose of an ACT team is to provide comprehensive and integrated services from one team so that service users should not need to access many services outside the team. The Norwegian legislation limits what various services can do. For example, housing for the ACT teams’ service users is the responsibility of the municipality, and the Labour and Welfare Administration is responsible for providing service users’ social benefits and pensions. This situation may interfere with the ACT teams’ work and opportunities to fully implement the model. An evaluation of ACT in Sweden found that administrative borders between authorities and the teams’ limited opportunities to intervene in the provision of services such as housing and social services were important obstacles [49]. Sweden also has a service system divided into two organizational levels and has similarities with the Norwegian system.

The staff in the Norwegian teams wanted to collaborate with other services even when it was not necessary under legal regulations; for example most teams collaborated with the primary services for home nursing such as medical delivery, which may be appropriate because of the long travel distances for some teams. Monitoring of the team members’ activities showed that 13% of contacts with service users included collaboration with other service providers [39]. Our fidelity assessment showed that the ACT teams assumed responsibility for services even when co-operating with other services. This meant that the service users did not need to access many services outside the team. Collaboration is needed in the co-ordination of different services, but co-ordination may be time consuming and may increase the risk that the services are not comprehensive.

The existing local service system is important to consider when implementing ACT as a new service delivery system. It should be well resourced but should not be an alternative model to ACT based on close integration of a full range of care. ACT should not compromise the functions of the existing services and should be supported by local stakeholders [50]. The Norwegian local service system is well resourced compared with that of many other countries, but it is not well integrated and has not incorporated the core elements of the ACT model. Even though the Norwegian ACT teams are based on co-operative agreements between the primary and secondary services, the teams discussed the distribution of roles and functions between various services (such as what different services should do) and the ACT teams sometimes intervened in the existing services. There were also disagreements about the target group when some stakeholders wanted the teams to include a broader group of service users. One important challenge for the Norwegian ACT teams was finding ways to co-operate with other services. Future work is needed to identify the best ways to adapt ACT teams within the rest of the mental health service system.

## Strengths and limitations

The study’s major strength is that the fidelity assessments using the TMACT instrument provided extensive data about the teams. The feedback from the teams indicated that the fidelity assessments provided reliable descriptions of the teams and that they found the fidelity assessment useful for their further development. Unfortunately we did not conduct inter-rater reliability tests. However, to prevent variations in scores because of different interpretations of the TMACT items, the research group of six persons underwent the same training before the study started. The TMACT manual also had explicit instructions of ratings to minimize the raters’ subjectivity of the scale and the research group had several meetings to discuss the use of the TMACT and discussed their ratings of all teams at both 12 and 30 months. The study included 12 teams operating in different parts of Norway, but these were not a representative sample of Norwegian communities because rural areas of the country were under-represented. All teams were newly established and were implementing a new model in the Norwegian context, which could have had both positive and negative influences. The teams’ staff were highly motivated, but they did not have all the necessary resources in place because they lacked skills and training in certain aspects of evidence-based treatment.

The focus group interviews complemented the fidelity assessments. They provided a broader range of information about the adaptation of the ACT model by the teams and valuable information about the practice of ACT in different Norwegian contexts. Focus groups have limitations with regard to dominant views. The ACT team members knew each other well and were used to discussions within the team. The impression from the interviews was that honest views were expressed in the various discussions. However, there is always a danger that some participants feel pressured to agree with dominant views or do not express their own opinion. The moderator tried to ensure that all main themes were covered, but the involvement in the topics differed between the teams according to the local situation. It was challenging to analyse a large volume of data for which the topics were given different weight. The retrospective perspective may also be a limitation because the data relate to the team members’ reflections after 30 months of operation of ACT.

## Conclusions

The ACT team members in Norway experienced the ACT model to be a good service delivery model for the target population. Collaboration with other health and social services in both municipalities and specialized health services and inclusion of ACT teams into the local service system were perceived as challenges by the team members. Important parts of the ACT model had high implementation (subscales of Operation and Structure and Core Team). The newer parts of the model related to recovery and evidence-based practices had moderate implementation, and the specialist functions had low implementation. In conclusion, this study demonstrated that the ACT model could be implemented throughout Norway without extensive adaptions even though it has been developed in a different context.

## Data Availability

The dataset used for analysing ACT fidelity during the current study is available from the corresponding author on reasonable request. The data from the focus group interviews are not publicly available for reasons of confidentiality.

## Abbreviations

ACT: assertive community treatment
FACT: flexible assertive community treatment
DACTS: Dartmouth Assertive Community Treatment Scale
TMACT: Tool for Measurement of Assertive Community Treatment
CMHC: community mental health centre
SD: standard deviation
